# Evaluation of Patient-Assessed Quality of Life Questionnaires Following Operative Treatment of Pelvic Fractures

**DOI:** 10.3390/jcm14197036

**Published:** 2025-10-04

**Authors:** Piotr Walus, Jakub Ohla, Benson Akinola, Michał Wesołowski, Jakub Bulski, Maria Zabrzyńska, Rafał Wójcicki, Tomasz Pielak, Bartłomiej Małkowski, Jan Zabrzyński

**Affiliations:** 1 Department of Orthopaedics and Traumatology, Faculty of Medicine, J. Kochanowski University in Kielce, 25-001 Kielce, Poland; 2 Department of Orthopaedics and Traumatology, Faculty of Medicine, Collegium Medicum in Bydgoszcz, Nicolaus Copernicus University in Toruń, 85-092 Bydgoszcz, Poland; 3 Department of Physiology and Pathophysiology, Division of Pathophysiology, Wroclaw Medical University, 50-368 Wroclaw, Poland; 4 Department of Urology, Oncology Centre Prof. Franciszek Łukaszczyk Memorial Hospital, Bydgoszcz, dr I. Romanowskiej St., 85-796 Bydgoszcz, Poland

**Keywords:** WHOQL, SF36, pelvic fracture, quality of life, acetabular fracture, pelvic injury

## Abstract

**Objective:** To evaluate patient-assessed quality of life (QoL) following operative treatment of acetabular fractures and pelvic ring injuries and identify differences in their assessment of QoL depending on sex and age group. **Methods:** The study included 75 patients, 41 who had suffered acetabular fractures and 34 pelvic ring fractures, who had been treated operatively over a 6-year period (2017–2022). Post-operative HRQOL scores were evaluated using the World Health Organization Quality of Life BREF scale (WHQOL-BREF) and RAND Short Form 36 (SF-36). Separate analyses were completed for men and women, and we compared the data between five age groups. **Results:** The male patients scored slightly higher in all domains than the women in the acetabular fracture group. In the ring fracture group women reported higher scores in the psychological (72.67 vs. 69.44) and social domains (81.67 vs. 77.08). The men (80%) reported more significant overall satisfaction with their health in the acetabular group compared with the women (*p* = 0.0306). In the SF36 analysis, for both acetabular and ring fracture groups the lowest average QoL was recorded in the physical health composite summary (PHC) with a score of 41.34 ± 9.49 and 41.21 ± 9.19, respectively. Men scored higher for all eight scales in the ring fracture and all except general health in the acetabular fracture group (*p* = 0.0166). For the mental health composite (MHC), men had a better mean score in both fracture groups with significant differences for between both genders for the acetabular group (*p* = 0.0352). For age group analysis of the SF36, in the acetabular group, the youngest age group (<40 years) reported a significant decline in their performance due to role limitations due to physical health (RP) and emotional problems (RE) compared with the oldest group (>70 years) (*p* = 0.0306 and *p* = 0.0069, respectively), similarly to the PHC (*p* = 0.0279). Additionally, for the overall mental health summary of the acetabular group for the five age groups, there were significant differences between the youngest age group and the oldest age group (*p* = 0.0372). In contrast, for the ring fracture group, the oldest age group (>70 years) scored the worst in all four scales of the physical health composite and the <40 years patients had the highest score for the physical functioning (PF) scale, with statistical significance when compared with the oldest group (85 (17.53), *p* = 0.01501). Additionally, the lowest mean score for the PHC was recorded in >70 years and the highest in the 61–70 years age group, with a statistical significance (*p* = 0.0367). **Conclusions:** Patients that sustain a pelvic fracture and are treated operatively are at a higher risk of deterioration in quality of life. Using both functional assessments, male patients emphasized improved quality in more spheres that was evaluated than women.

## 1. Introduction

The health-related quality of life (HRQOL) in patients after trauma or any disease is essential in the estimation of the efficacy of the therapy employed. The impact of a disease on a patient is not limited to its incidence within the general population, or the therapeutic approach. It is also important to measure or estimate the various dimensions for the quality of life (physical, emotional, social function, spiritual, psychological, etc.) of the patient. Therefore, in these orthopedic, non-orthopedic, and traumatic injuries it becomes important to review the health-related QOL measurements by implementing these tools at specific pre- and post-operative time points. The results can inform on improving the parts of the treatment algorithms that add real value to the patient’s understanding of the prognosis [1].

Pelvic injuries are often high energy traumatic injuries that have high morbidity and disabling features. These include, and are not limited to, pelvic ring fractures and acetabular fractures. Pelvic fractures are highly predominant in developing countries due to a high rate of road accidents and falls. Many of these pelvic fractures tend to be stable injuries, but sometimes unstable and exposed pelvic fractures increase the risk of mortality [2,3]. Management is often surgical and after this, it is important to evaluate the health-related quality of life (HRQOL) for the long-term evaluation of the patients after surgery.

Various functional scoring systems have been used for analysis of the quality of life in patients following pelvic ring and acetabular fractures. These include the Short Form (SF)-36 [4], World Health Organization Quality of Life BREF (WHOQOL-BREF) score [5], Musculoskeletal Function Assessment (MFA) questionnaire [6], Life Satisfaction Scale (Lis-AT 11) [7], EuroQol Quality of Life Score (EQ-5) [8], and others. These instruments have been standardized and validated for the subjective evaluation and objective interpretation of their results. However, it has been reported that there are no specific questionnaires to assess treatments of acetabulum and pelvic fractures; hence, several traditional hip scores for arthroplasty patients are combined with them [9,10,11].

Several studies have reported controversies regarding the prediction of HRQOL in pelvic fractures. It has been presented that age and sex are not factors in the outcome, however, few authors reported a significant relationship to the outcome [12,13,14,15]. On the other hand, different studies stated that age stands as an independent risk factor of HRQOL in geriatric patients following pelvic fracture [16], and the degree of residual displacement is a significant predictor of HRQOL [17].

Functional outcomes following pelvic fractures are also dependent on the associated visceral and soft tissue injuries in pelvic fractures [17,18]. The severity of these pelvic fracture-related associations often contributes to a poor functional outcome even with or without the contributions of instability and asymmetry, but this could be due to severity and the amount of damage to the soft tissues [19]. In the study by Michaels et al. it has been shown that patients with orthopedic injuries scored worse than patients without orthopedic injuries in six of eight SF-36 domains following blunt trauma [20].

The study by Borg et al. with a mean patient age of 49 years using SF36 as one of their assessment tools showed a positive correlation between the quality of fracture reduction and patient’s health-related outcomes [11]. This contradicts Miller et al.’s study of elderly patients (mean age of 67 years) with acetabular fractures, which found no correlation between functional outcomes and the quality of the reduction [21].

The aim of this study was to evaluate the patient-assessed quality of life of patients following operative treatment of an acetabular fracture and pelvic ring injury in our trauma center between 2017 and 2022. To achieve this, we employed the WHOQOL-BREF and SF-36 for male and female genders across five age groups.

## 2. Material and Methods

### 2.1. General Characteristics

**Scheme 1:**
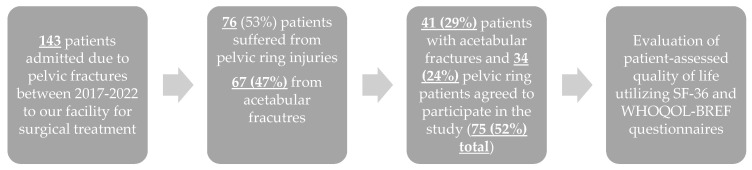
Presentation of the enrollment process.

The study included patients operated on due to an acetabular fracture or pelvic injury in our center in the years 2017–2022. We had implemented the following inclusion criteria: acetabular fracture or pelvic ring injury treated operatively, operative treatment in our facility between 2017 and 2022. Acetabulum fractures were classified according to the Judet and Letournel classification (anterior column with posterior hemi-transverse fracture (AT), anterior column (AC), both columns (BC), posterior column (PC), posterior column  +  posterior wall (PC + W), posterior wall (PW), transverse (T), transverse with posterior wall fracture(T  +  P)). Pelvic ring injuries were classified according to the Young and Burgess system (anteroposterior compression (APC), lateral compression (LC), vertical shear (VS), and combined mechanisms (CMs)). Exclusion criteria: conservative treatment of acetabular fracture or pelvic injury. Interviews were conducted by two medical doctors from our facility in August 2024. Patients completed the surveys via telephone. Scheme 1 represents the enrollment process.

The interviews consisted of two questionnaires: the Short Form 36 (SF-36) and World Health Organization Quality of Life BREF scale (WHQOL-BREF). The Medical Outcomes Study 36-Item Short Form Health Survey (SF36) is a self-administered standardized method for estimating the status of a patient’s health. It comprises 36 items or questions that measure general overall well-being and functional status. SF-36 is a popularly used tool in the assessment of health in various studies. WHOQOL-BREF scores are individualized scores that aim to encompass every possible aspect of a patient’s being, to evaluate the ultimate outcome of any intervention for a disease. In measuring HRQOL, the WHRQOL-BREF takes the view that it is important to know how satisfied or bothered people are by important aspects of their life, and this interpretation will be a highly individual matter. WHRQOL assessment—the WHRQOL score is a cross-culturally valid assessment of well-being and is available in most of the world’s major languages. Complete questionnaires are provided in the Supplementary Materials.

### 2.2. Description and Explanation of the Questionnaires

#### 2.2.1. WHOQOL-BREF

To perform this objectively, the short version of the QoL of the World Health Organization questionnaire (WHOQOL-BREF; Polish translations) was employed. The questionnaire informs of the quality of life by scoring four QoL domains: physical health, psychological health, social relationships, and environment. Quality of life for the last 4 weeks was based on the questions within the WHOQOL-BREF questionnaire and was analyzed. The WHOQOL-BREF questionnaire consists of 26 items scored on a five-point scale ranging from lowest (1) to highest (5) except for the last question (item 26), where 5 is a negative observation and 1 is the better score. In total, 24 items are included to calculate the final scoring of each domain, but global item 1 (What is your quality of life?) and item 2 (Are you satisfied with your health?) are scored separately. The total score for each domain is calculated and provided, with a higher score indicating a better quality of life from the respondent.

#### 2.2.2. SF-36

For the measurement of quality of life, another useful instrument frequently used is the Short Form 36 (SF-36), developed in the Medical Outcomes Study (MOS) by Ware et al. [22]. SF-36 informs only of participants’ morbidities. Unlike the WHOQOL, the SF-36 consists of 36 questions that are scored based on responses indicated within the questionnaire with equivalent numerical substitutes [23,24]. Additionally, of the 36 questions, 35 are used to create eight domains of life assessment that includes physical functioning (PF), bodily pain (BP), role limitations due to physical health problems (RP), role limitations due to personal or emotional problems (RE), general mental health (MH), social functioning (SF), energy/fatigue or vitality (VT), and general health perceptions (GH). Question 2 in the SF-36 is not used in the 8 domains mentioned previously, but it simply indicates the general health change based on the individual’s opinion within the last one year.

The original responses for each question are between 0 and 6 depending on the response category. In the scoring for example, item 20 with a possible 5 choice response has a high score (5) indicating a negative outcome of the respondent activity and a low value (1) meaning no limitation in social activities. In contrast, for item 32, the same response is inversed, i.e., 1 means there is a limitation and 5 means the best possible outcome. Therefore, there is a need to re-code the values recorded. Each domain contains a specific number of items and average if each re-coded outcome constitutes the final value, with an averaged higher value indicating higher or better functioning. The scores are standardized so that each item of the questions is within a range of 0 to 100, i.e., the minimum is 0 and the maximum value of any domain is 100.

The physical function has 10 questions (3–12); role limitations due to physical health has 4 questions (13–16); role limitations due to emotional problems has 3 questions (17–19); energy/fatigue has 4 questions (23, 27, 29, 31); emotional well-being has 5 questions (24, 25, 26, 28, 30); social functioning has 2 questions (20, 32); bodily pain has 2 questions (21, 22); and general health has 5 questions (1, 33–36). Finally, the domains can be summarized into a physical component summary (PCS) and a mental component summary (MCS), using population normal weights and the Z-scores of each scale of the SF-36 to derive a summary. The calculations, assumptions, and shortcomings for the MCS and PCS have been discussed and presented in several studies [25].

### 2.3. Ethics

Permission to conduct the following study was obtained from the Nicolaus Copernicus Bioethics Committee in Torun (approval number KB 645/2022). Informed consent was obtained from all patients or their legal guardians to include them in scientific studies.

### 2.4. Statistical Analysis

The measured data were processed and statistically evaluated with Excel software (Microsoft Corporation 2024, office.microsoft.com/excel) and NCSS 2023 Statistical Software (NCSS, LLC., Kaysville, UT, USA, ncss.com/software/ncss). The descriptive statistics of numerical variables, i.e., frequency count, mean, standard deviation, and range, were used to summarize the data. For comparison of the variables, within the groups, each outcome was measured and the nonparametric tests (Kruskal–Wallis Test and Mann–Whitney U test) were performed depending on the normality of the data. Statistical significance was, for a *p*-value, < 0.05. The Spearman correlation coefficient was calculated to determine the correlation of the different subscales of the SF-36.

## 3. Results

### 3.1. WHOQOL-BREF

#### 3.1.1. Acetabular Fractures

For the acetabular fracture group, the analysis of the QoL domain scores after transformation of the data is presented in Table 1. There are total of 41 patients (men: 33, and women 8) who were analyzed. The lowest score was recorded for the physical health domain with a mean value of 58.62 (10.13) and the highest mean score of 80.95 (11.54) was recorded in the environmental domain.

The results for the global items were compared for the acetabular group, and there was a statistically significant difference (*p* = 0.034) between observations on how the respondents felt about their health post-surgery and their quality of life for the last 4 weeks. A mean value of 3.34 was recorded for the quality of health; however, there was an increased average value for the post-surgery quality of life at 3.81.

The comparison within sex for the acetabular group for all four domain is shown in Table 2. There was no significant difference between the sexes for the QoL in the physical health (*p* = 0.5595), psychological health (*p* = 0.4953), social relationship (*p* = 0.5599), and environment (*p* = 0.8042) domains, respectively.

Similarly, for global quality of life, there was no significant difference (*p* = 0.7205). However, for quality of life with respect to how the patients were satisfied with their health, the females’ group of acetabular fracture had a mean value of 2.75, and the male group had a bigger average value of 3.485, with a statistically significant difference indicated between both groups (*p* = 0.0306).

A comparison was made for the impact of the various domains of QoL on age. The patients were divided into five age groups as shown in Table 3. For patients aged 40 and less, there was no significant difference in physical health when compared with those in the age groups of 41–50, 51–60, 61–70, and greater than 70 years of age. Similarly, comparing all age groups for the psychological health, social, and environmental domains, there were no significant differences.

Therefore, age did not impact the perceived QoL. Additionally, for the global items, quality of life (*p* = 0.95) and satisfaction with health (*p* = 0.967) were not significantly different across the age groups.

#### 3.1.2. Pelving Ring Injury

The mean level for the physical health domain was 56.62 with a standard deviation of 10.37 and a range of 35.71 to 71.43. Likewise, the psychological domain had a higher mean value for all respondents with a mean of 70.10 (10.13) and ranging from 50.00 to 83.33. The social domain had a mean value of 78.43 (18.93) and range of 33.33–100. The environmental domain had similar values at a mean of 79.51, standard deviation of 11.92, and range between 56.25 and 100. Similar, to the pelvic fracture group, the physical health domain scored the lowest of all four domains. The given results are presented in Table 4.

A comparison across gender for the four domains is shown in Table 5. For all domains, there were no significant differences in the responses by females and males for the physical health, psychological, social, and environmental domains. The responses for quality of life and health satisfaction are also presented in the table. The mean value for satisfaction of health within the female group was 3, with a standard deviation of 1.25. In the male group, this was a mean value of 3.58 and a standard deviation of 1.06. However, while women reported less satisfaction, based on these results, the difference between each group was not significantly different for quality of health, with a *p* = 0.2212.

The age intervals <40, 41–50, 51–60, 61–70, and >70 years were used to group the respondents to evaluate the scoring for the quality of life across the different age groups as presented in Table 6. There were no statistical differences across all 4 domains for all age group divisions. In the physical health domain, the 61–70 years age group had the highest mean value of 62.86 (6.96). Respondents less than 40 years of age had the highest mean value for the psychological domain with a mean of 74.48 (11.66) and in the environmental domain with a mean if 84.77 (13.09). In the social domain, the age group > 70 years had the biggest mean value of 88.33 (7.46).

Typically, the domain scores can be presented as a scaled mean score and should be within a range of 4 to 20. The results for both groups are presented in Table 7.

### 3.2. SF-36

#### 3.2.1. Acetabular Fractures

In the analysis of the acetabular group fracture, the mean for the RAND-36 scores for the eight scales and the composite for the physical and mental health domains were calculated for the group and for the gender categories for males and females in the group (Table 8). The mean (standard deviation) of age was 55.73 (16.25) years with a range between 29 and 86 years. All scales scored above 50 except for general health, which was 47.2 (4.62). There were more men in the acetabular injury group with a mean age of 56.82 (14.7) years, and women had a mean of 51.25 (22.17) years. The men had better scores in all scales aside from general health (47.12 (4.51) versus 47.5 (5.35)). Women responded to exhibiting worse social functioning, and there was a significant difference between the men and women of 90.53 (23.81) and 64.06 (33.03), respectively (*p* = 0.0166).

The women scored poorly for the physical and mental health composite with scores of 38.02 (10.82) and 39.98 (11.67), respectively. The same was seen in the male group with scores for both less than 50. However, there were significant differences between the overall mental health of both gender groups (*p* = 0.0352), where women had a worse overall score compared with men, 39.98 (11.67) and 49.14 (10.1), respectively.

The patients were divided into 5 different age groups to measure the differences across the scales (Table 9). For the analysis of the four scales contributing to physical health (PF, RP, BP, and GH), the youngest group (<40 years) had worse scores for physical limitations, pain, and general health and had substantially reduced energy. In contrast, the oldest group reported the best scores in reduced limitation in physical health and reported less pain. The age group 41–50 years had the highest values for general health (though still lesser than 50 points, i.e., 48.57 (6.9)), although all age groups had roughly equal scores for general health. There was no statistical difference in all age groups for the difference in mean rank for all 4 scales. However, when pair-wise comparisons were made between the age groups, a significant difference was seen between the <40, 51–60, and >70 years age groups for physical role functioning. This means that in the measure of the limitation of how patients performed various roles of work and daily activities, the youngest group reported a significant decline in their performance compared with the oldest group, who reported a much better score (*p* = 0.0306). Additionally, this difference was seen in the overall summary, with statistical significance seen for the PHC (*p* = 0.0279). For the four scales of the mental health composite, the youngest group scored the lowest for VT, SF, RE, and MH. Those aged 41–60 years reported the highest values for energy and social functioning. The >70 years group had the best scores for role limitation due to emotional problems and emotional well-being. Statistical differences between groups were seen between the oldest and youngest group for RE (*p* = 0.0069). There was fairly no difference between the scores reported for those between 51 and 70 years for level of fatigue/energy and emotional well-being. Additionally, there was a significant difference between the youngest group (<40 years), and the 60–70 age group (*p* = 0.01337) and when compared with the oldest age group (>70 years, *p* = 0.0372) for the mental health composite.

There was a correlation between age and the eight scales with the two overall composites for mental and physical health, as shown in Table 10. There was no significant difference measured between the patient’s age and the scales, with very weak correlations recorded.

For inter-scale (i.e., between the majorly contributing scales of physical and mental health) correlations (Table 11), the weakest correlation was between general health and role limitation due to emotional problems (r = 0.12), while emotional well-being and energy/fatigue had the strongest correlation (r = 0.72). For within the physical health scales (PF, RP, BP, and GH), there was a correlation range of between 0.43 and 0.7, with the correlation between body pain and physical functioning the highest (r = 0.70) for physical health scales. Similarly, for the mental health scales (VT, SF, RE, and MH), the correlation range was between 0.21 and 0.72, with the correlation between energy/fatigue (and emotional well-being) versus role limitations due to emotional problems being the lowest (r = 0.21).

#### 3.2.2. Pelvic Ring Injury

The ring fracture group consisted of a total of 34 patients with a mean age of 51.09 (15.99) years that included 10 females and 24 males having an average age of 56.30 (18.96) and 48.92 (14.47) years, respectively. For the eight categories accessed using the SF-36 questionnaire, the role limitations due to physical health (RP) and general health (GH) scored very poorly (<50) with 49.26 (46.66) and 45.44 (5.13), respectively. In contrast, patients felt that there was reduced limitation in social activities due to emotional problems, with a highest average point of 81.99 (26.86).

The average score and ranges for the age of the patients and the scales to predict quality of life is presented in Table 12. Furthermore, the estimated physical health component [41.21 (9.19)] and mental health component [47.90 (10.76)] also scored poorly. With a normal average value of 50 points, this indicates that while patients were happy with their physical health after surgery, they felt dissatisfied with their mental health.

To predict inter-patient differences, comparisons between gender in the subscales and composite data have been collected and are presented in Table 13. From the data, there were more men (70.59%) than women (29.41%) in the ring fracture group. The female group had a mean age of 56.3 years with a difference of more than 8 years from the male group (48.92 years). The physical health component in the female group was very poor with an average value of 38.06 (10.62), and the men had a higher score of 42.52 (8.42); however, there was no statistical differences between them (*p* = 0.1058).

For physical functioning, role limitation due to physical health, and general health, the male patients had high scores of 78.75 (19.90), 53.13 (46.31), 60 (22.12), and 45.63 (5.58), respectively. Additionally, the male group also scored more in the mental health composite with a value of 49.88 (10.65) and in all its contributing scales of mental health, i.e., VT (61.46 (20.24)), SF (85.42 (25.98)), RE (68.06 (42.25)), and MH (74.17 (15.06)). However, there were no significant differences between both gender groups for all scales.

The ring fracture group was also investigated across five (5) age groups and the descriptive statistics are shown in Table 14 with respect to the eight subscales of the Short Form. There were no female patients in the 50–60 age group. The age group between 61 and 70 years had the best scores in all scales and in the MHC and PHC against all age groups. However, this group still reported poorly on the physical health composite with a score of 48.69 (7.7) and that of general health at 48 (4.47). In contrast, the age group greater than 70 scored the worst in all scales contributing to the physical health composite.

There was no statistical significance between all groups. However, comparisons between the group with the highest mean values for each scale and the age group with the lowest showed statistical significance. In physical functioning, the less than 40 age group had the highest score of 85 (17.53), and there was a statistical difference between the group and those greater than 70 years (*p* = 0.01501). The age group 61–70 had the highest values in RP, BP, and GH, where >70 years scored the lowest; there was a significant difference only in the perception of body pain for both groups (0.0333). It should be noted that there was also a significant difference (*p* = 0.04653) when physical function between both groups was analyzed. Finally, comparing the PHC, there was a significant difference between group 61–70 and the oldest group (*p* = 0.0367). However, while it was not statistically significant, comparing all groups, the (61–70) age group performed best and was the only group to have a mean value of >50 in the mental health composite with a mean value of 51.79 (6.74).

The correlation between age and the eight scales and the PHC and MHC was calculated and is presented in Table 15. There was no statistical significance recorded.

For the correlation between the physical and mental health scales (Table 16), the strongest correlation was observed between emotional well-being and energy/fatigue (r = 0.78), and the weakest correlation was between social functioning and physical functioning (r = 0.36). For scales that make up the physical health composite (PF, RP, BP, and GH), there was a correlation range between 0.49 and 0.62, with the correlation between body pain and role limitations due to physical health (RP) the highest (r = 0.62) for the physical health scales. Similarly, for the mental health scales (VT, SF, RE, and MH), the correlation was strong and ranged between 0.55 and 0.78 with the correlation between energy/fatigue and role limitations due to emotional problems being the lowest (r = 0.55).

Role limitations due to physical health (RP) from the physical health scales had the strongest correlation with the other seven scales (range 0.48 to 0.73). Its lowest (r = 0.48) and highest (r = 0.73) was with energy/fatigue and role limitations due to emotional problems, respectively. Similarly, within the mental health scales, role limitations due to emotional problems (RE) correlated strongly with the other seven scales with a range between 0.57 and 0.71 and with its highest t correlation with social functioning (r = 0.71) (Table 17).

## 4. Discussion

### 4.1. Pelvic Ring Injury

Oliver et al. [26] analyzed the quality of life by administering the SF-36 questionnaire to 46 patients who had suffered from an unstable ring fracture after 16 months of injuries. They reported that physical activities were affected more due to the injuries with a measured 14% impairment in physical outcome and 5.5% impairment in mental outcome scores when compared with the general USA population norm. They compared for either Orthopedic Trauma Association (OTA) type B or C pelvic ring disruption and reported higher SF-36 values for both types of pelvic fractures. A mean PCS of 68.7 ± 27.6 and MCS of 72.2 ± 26.0 were recorded for type B and for type C, the mean PCS was 62.67 ± 25.8 and the MCS was 69.3 ± 25.06. Despite these higher values, in contrast, Lefaivre et al. [27] reported that there was no statistical difference for the MCS and PCS between type B and C pelvic fractures. Lefaivre et al. also reported more beneficial results in the B group with significant differences between both OTA B and C ring fracture types for the mental composite only but not for the PCS [27]. The mean value for both groups combined was PCS (43.26 ± 1.95) and MCS (46.74 ± 2.00) and this compared closely with our results (PCS: (41.21 (9.19)) and MCS: (47.90 (10.76)).

Similarly, Borg et al. [28] disclosed a more favorable outcome in type B fractures with a significant difference in only the general health between type B and C fractures. They had reported the QoL of 54 patients with pelvic ring fractures for 2 years using the SF-36. Borg et al. reported lower scores than the reference Sweden population norm in all eight domains [28]. The closest domain to the norm was in general health; the highest mean score in their result was for social function (68 versus 57.79 in our data) and role physical was the lowest they recorded (38 versus 72.79 in this study). In this current study, for the ring fracture group, the highest was role emotional (81.99), with fatigue having the lowest mean score (45.44). In a 2-year follow-up assessment of 57 patients of type B and C unstable pelvic ring fractures, Suzuki et al. reported a lowered average SF-36 score compared with the Japanese population norm, with 13.4 point and 9.5 point difference for PCS and MCS, respectively [12].

Ponsford et al. [29] recruited and analyzed 113 patients for 2 years. There were poorer outcomes in all eight domains compared with the controls 1 year post-injury, with physical role having the lowest mean value of 28.8 (87 in the control group) [29]. At 2 years post-injury, there was no significant improvement in all domains between the groups and fracture types. The pelvic fracture patients still showed significant disabilities. This result was also like that of Borg et al. [28]. However, when comparing between year 1 and year 2, the patients showed significant improvements in the physical summary score but none in mental health [28,29]

Ayvaz et al., in a >2-year follow-up post-fracture study of unstable pelvic fractures treated with closed reduction and percutaneous fixation, reported a SF-36 comparable to that of the Turkish population norms. The average PCS was 81.3 and the mental score was 80.8. The difference between these scores and those we have reported are significantly different [30].

In 112 patients with pelvic ring fractures managed surgically or conservatively, Verma et al. presented that 48.23% of these patients had a similar physical functioning to the population norm with an average SF-36 PCS score of 47.71 (7.88). MCS was 49.20 (9.37) with 65.3% of patients at the same level as the population norm [17]. However, both physical and mental average values were comparatively low to the general population norm [17]. Also, for different treatment types (i.e., operative and non-operative), Höch et al. reported that the mean PCS value was 44.8 ± 10.0, and this was lower than the average German population norm, and the mean MCS of 52.6 ± 15.0 was comparable to the population score [31]. The authors did not note any statistical difference when they compared both treatment groups. It should be noted that they excluded patients more than 65 years and those with pathological fractures [31]. However, when compared with our data for age groups less than 62 years, only the patients less than 40 years had similar mean values in the PCS (43.54 (8.02)). For the mental composites of all groups in our data, under 61 years of age scored lower compared with their mean values.

In the comparison across time points, Petryla et al. had also compared the quality of life of 32 patients aged between 18 and 65 years, after B2 pelvic-ring fracture fixation treatments (posterior fixation versus anterior + posterior fixation). The time point was firstly at hospitalization (about their pre-trauma state), and then secondly, 1 year later (postinjury). For within treatment group analysis, they reported post-injury time point median values with lowered physical health one year after pelvic surgery. PCS after 1 year was 49.1 (39.7–56.3) in the posterior fixation group and 48.4 (range 36.1–55.5) in the anterior + posterior fixation group, with both scores statistically significantly lowered after 1 year for both groups compared with the first hospitalization. However, there was no statistical difference for the mental health for time point and for the treatment approach type. This was a similar result to Lefaivre et al, who had recorded a mean value of 45.01 ± 2.36 for the PCS and 48.76 ± 2.54 for the MCS; however, Lefaivre et al. reported that although it was a favorable outcome for type B fractures, it was only statistically significant for the mental composite but not for the PCS [27].

### 4.2. Acetabular Fracture

Hernefalk et al. investigated the QoL for patients treated for both acetabular and pelvic fractures and reported a significant difference in the amount of bodily pain the patients described between both fracture groups [32]. Similarly, with a median age of 52 years in the patients investigated, the younger patients (less 52 years) had higher values in role physical and general health, with no statistical differences in other domains. In comparison of functional outcome with time point (1 month post-surgery and 2 months post-surgery), only the general health domain statistically significantly improved at 1 month post-surgery, and four of the eight domains (BP, GH, VT, and SF) were significantly higher at 2 months when compared [32].

In a different study, Borg et al. compared the quality of life over 2 years at different post-operative time points (6 months, 12 months, and 24 month) following internal fixation of acetabular fractures. The patients scored low in the QoL for both physical and mental domains of the SF-36 compared with the Sweden reference norm population [11]. However, they reported improvements from 6 months at 12 months and 24 months in physical function and physical role domain while other domains had no significant changes [11]. At 6 months, our data compared similarly with theirs in five of the subscales: physical function (50 vs. 67.93), role physical (0 vs. 54.27), bodily pain (52 vs. 57.68), vitality (55 vs. 56.22), and social function (75 vs. 85.37) [11]. Subsequently, we recorded lower values in general health (72 vs. 47.24), role emotional (100 vs. 71.54), and mental health (80 vs. 66.34) compared with our acetabular group. Also, similar numbers were obtained when compared with their results at 24 months.

Similarly, in the treatment of acetabular fractures, Patil et al. compared the functional outcomes using three surgical approaches: Kocher–Langenbeck, iliofemoral, or modified anterior intrapelvic (Stoppa) approaches [33]. They reported the SF-36 for 1, 2, 3, 6, and 13 months post-operatively. There was no statistical significance between the means of all three surgical groups, and the PCS scores were lower in the third month for all three groups but increased in 1 year [33]. The authors reported that the mean MCS score was highest in the Stoppa group and decreased in the iliofemoral and Kocher–Langenbeck groups at 12 months (though it increased for the two latter groups at month 3) [33].

Anglen et al. evaluated the functional outcomes in patients greater 60 years who had been surgical treated for acetabular fractures [9]. The average age of patients was 71.6 years and the follow-up was up to 37 months. They reported that the SF-36 domains were all within one standard deviation of the means of the age-matched USA population norms [9]. The mean value of the MCS (57.38) and its components scored slightly above the norm values, but the PCS (37.26), physical function, and role physical scored below the age-matched population normal means. The PCS was comparable with the one in this paper for the age group 61 to 70 (39.54 (8.29)); however, our MCS was significantly lower with a mean value of 45.94 (9.63).

### 4.3. Age

Holstein et al. [16] also signified that age was an independent predictor of the quality of life of pelvic-fracture patients. Verma et al. reported that the significance of age and gender was dependent on the degree of residual displacement. Gender and age did not influence the HRQOL for both WHOQOL-BREF and SF-36 for the residual displacement of less than 1 cm in their study [17].

In this study, the age group between 61 and 70 years had the highest score for both the mental and health composite for pelvic ring fractures.

### 4.4. Gender

In the investigation of the relationship between physical and mental functional outcomes in traumas, Holbrook et al. reported worse outcomes women [34]. Therefore, it was important to investigate the assessment of QoL, especially as it related to both mental and physical outcomes when investigating pelvic-specific measures [34].

For gender comparisons within both pelvic and acetabular fracture groups in Hernelfalk et al.’s study, the males had higher scores in vitality and social function domains only [32]. For the comparison between gender, Lefaivre et al. similarly did not report any significant differences between males and females for all scales of the SF-36 [27].

In our study, for ring fractures, though there were more favorable outcomes for males in all scales, PCS and MCS, these differences were not statistically significant for our representative study group. Similarly, in the acetabular fracture group, the male patients had better scores in seven of the eight scales with a statistical difference in social functioning when compared with the female group. The MCS was also statistically significantly improved in men (49.14) than in women (39.98).

### 4.5. Comparison to Different Populations

In the study for the quality of life in a representative polish population, Jaracz et al. assessed 908 patients [35]. In the data, they presented the mean score without any transformation. The mean values were 14.39 (2.82), 13.13 (2.62), 14.09 (3.14), and 12.91 (2.41) for the physical, psychological, social, and environmental domain, respectively. The results in our study were higher than theirs in the last three domains and lower for the physical domain (13.38 (1.62) in acetabular and 13.06 (1.66) in pelvic ring fracture). Similar, in psychometric testing of the Norwegian population by Hanestad et al. including 4000 Norwegian citizens, aged 19 to 81, the four WHOQOL-BREF domain mean scores were 15.78 (2.79) for physical, 15.16 (2.40) for psychological, 14.93 (2.69) for social, and 15.27 (2.40) for environmental domains [36]. Both acetabular and pelvic results were lower in the physical domain but compared equally or higher in the remaining three domains [36].

In the WHOQOL-BREF data of the Australian population norm reported by Murphy et al., the population norm was 80 (17.1) in the physical domain, 72.6 (14.2) in the psychological domain, 72.2 (18.5) for social relationships, and 74.8 (13.7) in the environment domain [37]. For our data, we reported higher mean values in social relationships (77.03 (17.46) in acetabular and 78.43 (18.93) in pelvic ring fracture) and the environmental domain (80.95 (11.54) in acetabular and 79.51 (11.92) in pelvic ring fracture) compared with the Australian norm; however, in the physical health and psychological domains and the two global items (QoL and health), we had lower mean values. Similarly, comparing with the Danish healthy population reported by Nørholm and Bech, with domain mean scores of 88.9, 78.1, 74.6, and 80.3 in physical, psychological, social, and environmental domains, respectively [38], our results were lower for both the physical and psychological domains for both acetabular and pelvic ring fracture groups; however, our results were similar for the remaining two domains.

In the study observing the functional outcome of operatively treated acetabular fractures over a period of 14 years, Meena et al. reported a domain mean score of 63.06 ± 20.31 (vs. 58.62 (10.13) in our acetabular group), 58.22 ± 19.57 (vs. 69.92 (11.16) in our acetabular group), 70.49 ± 17.92 (vs. 77.03 (17.46) in our acetabular group), and 64.48 ± 18.46 (80.95 (11.54) in our acetabular group) in the physical, psychological, social, and environmental domains, respectively [39]. They reported that these outcomes were influenced by associated injuries, delay of surgery, and the quality of reduction. Also, their result was comparable to the Indian general healthy population in the social and environmental health domain but was lower for physical and psychological health [39]. Compared with our study, we recorded high values in three of the four domains.

In the study of the functional outcome and health-related quality of life (HRQOL) after pelvis fractures in 112 patients, more than 50% of the patients achieved the general Indian population norms for the four domains of the WHOQOL-BREF [17]. They recorded lower numbers versus the general population norm in the physical (66.57 ± 20.46) and psychological domains (60.04 ± 20.04) and higher mean values of 70.54 ± 20.56 and 74.33 ± 16.74 in the social and environmental domains, respectively [17]. In comparison with the data in this study, our patients in the ring fracture group scored lower in the physical health domain at 56.62 (10.37) but had a high mean value for each of the remaining three domains: 70.1 (10.13) in the psychological domain, 78.43 (18.93) in the social domain, and 79.51 (11.92) in the environmental domain. Verma et al., recorded no significant impacts of age and sex on all domains, and this was the same for our results for both pelvic ring fractures and acetabular fractures [17].

In the study presenting the analysis of the Polish population for the Polish version of the SF-36 questionnaire, developed by Żołnierczyk-Zreda, the author presented that for the total of 823 people, the population norm for the physical and mental health composites were 48.55 (9.80) and 49.30 (11.06) for the entire group [40]. While we did not have a total population, our acetabular group had similar scores and were also less than 50, with the physical health and mental health composites much lower than the Poland population norm, at 41.34 (9.49) and 47.35 (10.91), respectively. However, for the ring acetabular group, we reported a significant difference compared with the polish norm with a reported physical health component of 71.53 (14.5); however, like the whole polish group, mental health was 41.21 (9.99) [40].

Additionally, for gender differences, the mean value for the physical and mental health component of men were 48.82 (10.56) and 49.50 (10.93) in the polish population norm. While the mental health component in the acetabular group (49.14 (10.1)) and ring fracture group (49.88 (10.65)) compared very closely to the polish norm, the physical health components were less than the population norm for the acetabular group (42.14 (9.14)) and ring fracture group (42.52 (8.42)). The physical health and mental health components for the females were 48.37 (9.27) and 49.16 (11.16), respectively, and when compared with the females in the acetabular group (38.06 (10.62) and 43.15 (9.94)) and the ring fracture group (38.02 (10.82) and 39.98 (11.67)), they were remarkably different compared with those of the population norm, especially for the ring fracture group.

When comparing with the US norm, Blanchard et al., 2004 [41], reported that for the RAND-36, a physical health composite score lower than 43 and mental health composite (MHC) score of less than 39 meant poor physical and psychological health. However, mean values greater than 53 in both the PHC and MHC were indicative of improved quality of life [41].

## 5. Limitations

Michaels et al. reported that orthopedic patients with blunt traumas scored worse than non-orthopedic patients in six domains of the SF-36 after 1 year of surgery [20]. Several patients of pelvic traumas sustained multiple injuries involving the abdomen, spine, extremities, thorax, and head/neck (Höch et al., Rainer et al.), with high rates of associated injuries in pelvic-fracture patients (Failinger & McGanity, Verma et al.). Hence, there is a question of the estimation of the additional concomitant injuries and their influence on the quality of life [17,31,42,43].

These concomitant injuries altered the long-term outcome of patients with unstable pelvic injuries (Ayvaz et al. Kabak et al., Suzuki et al.) [12,30,44]. The influence of associated injuries was highlighted with patients who had no associated injuries having higher statistically significant mean physical function scores (Borg et al.) [11]. However, Verma et al., in their study, reported that additional and associated injuries or the manner of injury did not have a significant effect on the QoL, but they noted that this was dependent on the limit of residual displacement [17]. Hence, this was one of the limitations of this study since we did not investigate or compare the QoL with results from pelvic-specific instruments like the Majeed Score (Majeed 1989) or Iowa Pelvis Score (IPS) (Templeman 1996) [45,46].

There is no established guideline on comparing or analyzing the functional outcome in terms of self-assessed QoL of pelvic trauma patients with the general population norm. Additionally, in this study, we have only analyzed post-traumatic evaluation following surgical intervention. We are unable to compare with the pre-traumatic quality of life of the same patients. We investigated patients only from our trauma center that were treated operatively.

Additionally, we are unable to compare their QoL at various timelines after surgery to see if there is still improvement or deterioration.

There is also no influence or examination of associated injuries, and other sociodemographic factors including education, employment status, relationship, family, other health comorbidities etc.

## 6. Conclusions

Patients that sustained a pelvic fracture and were treated operatively are at a higher risk of deterioration of quality of life compared with a global population. Standard outpatient check-ups of patients with pelvic fractures should include an assessment of their psychophysical condition. In the case of this group of patients, the orthopedic surgeon should be aware of the possibility of mental health disorders requiring specialist care and the long-lasting deterioration of the quality of life and functionality.

There is a need to create dedicated questionnaires assessing the health status in this group of patients. Further studies are needed to assess the QoL of patients with fractures of the acetabulum and pelvic ring. These studies should be multicenter in order to create homogeneous groups of patients with large numbers, enabling the presentation of QoL prognosis for a specific type of fracture according to commonly used classifications, such as Judet and Letournel or Young and Burgess.

## Figures and Tables

**Table 1:** Acetabular fracture group QoL domain and global item scores.

Domain or Item	Number (N)	Mean (Standard Deviation)	Range
Domains			
Physical health	41	58.62 (10.13)	32.14–71.43
Psychological	41	69.92 (11.16)	37.51–87.52
Social	41	77.03 (17.46)	41.67–100.0
Environmental	41	80.95 (11.54)	56.25–100.0
Items			
What is your quality of life?	41	3.81 (0.90)	2.00–5.00
Are you satisfied with your health?	41	3.34 (0.99)	1.00–5.00

**Table 2:** Gender association with QoL in the acetabular group.

Variable	Female (*n* = 8)	Male (*n* = 33)	*p*-Value
Age	51.25 (22.17)	56.82 (14.7)	
Physical health	54.47 (15.36)	59.63 (8.45)	0.5595
Psychological	69.27 (7.02)	70.08 (12.03)	0.4953
Social	73.96 (18.06)	77.78 (17.51)	0.5599
Environmental	80.47 (12.25)	81.06 (11.56)	0.8042
What is your quality of life?	3.625 (1.19)	3.848 (0.83)	0.7205
Are you satisfied with your health?	2.75 (0.89)	3.485 (0.97)	0.0306 *

* Statistically significant relationship (*p* < 0.05).

**Table 3:** Impact of age on QoL in the acetabular group.

Domains	Age (Years)	Mean (SD)	Range	*p*-Value *
Physical health	<40	55.72 (12.84)	32.14–71.43	0.8309
	41–50	61.74 (7.35)	50–71.43	
	51–60	55.95 (12.91)	35.71–71.43	
	61–70	61.11 (6.55)	50–71.43	
	>70	58.73 (10.43)	42.86–71.43	
Psychological	<40	70.83 (10.76)	50–83.33	0.7786
	41–50	73.21 (9.27)	58.33–83.33	
	51–60	64.58 (13.11)	45.83–75.08	
	61–70	67.59 (15.42)	37.5–87.52	
	>70	72.22 (6.25)	62.5–79.17	
Social	<40	77.5 (22.58)	41.67–100.00	0.6434
	41–50	85.72 (13.36)	66.67–100.00	
	51–60	75 (17.48)	50–91.67	
	61–70	75 (16.14)	58.33–100.00	
	>70	73.15 (16.55)	50–100.00	
Environment	<40	79.07 (12.68)	62.5–90.63	0.9327
	41–50	81.7 (13.55)	56.25–96.88	
	51–60	82.82 (12.62)	65.63–96.88	
	61–70	79.17 (11.27)	62.5–96.88	
	>70	82.99 (10.26)	68.75–100.00	

* *p*-value based on Kruskal–Wallis test, SD—standard deviation.

**Table 4:** Pelvic ring fracture group QoL domain and global item scores.

Domain or Item	Number (N)	Mean (Standard Deviation)	Range
Domains			
Physical health	34	56.62 (10.37)	35.71–71.43
Psychological	34	70.1 (10.13)	50.00–83.33
Social	34	78.43 (18.93)	33.33–100.00
Environmental	34	79.51 (11.92)	56.25–100.00
Items			
What is your quality of life?	34	3.59 (1.10)	1.00–5.00
Are you satisfied with your health?	34	3.41 (1.13)	1.00–5.00

**Table 5:** Gender association with quality of life within the pelvic ring injury group.

Variable	Female (*n* = 10)	Male (*n* = 24)	*p*-Value
Age	56.30 (18.96)	48.92 (14.47)	
Physical health	55.36 (9.11)	57.14 (11)	0.5944
Psychological	71.67 (9.58)	69.44 (10.47)	0.6712
Social	81.67 (17.48)	77.08 (19.7)	0.6047
Environmental	78.44 (11.55)	79.95 (12.29)	0.6757
What is your quality of life?	3.3 (1.34)	3.71 (1.0)	0.4418
Are you satisfied with your health?	3 (1.25)	3.58 (1.06)	0.2212

**Table 6:** Impact of age on QoL in the ring fracture group.

Domains	Age (Years)	Mean (SD)	Range	*p*-Value *
Physical health	<40	55.36 (13.23)	35.71–71.43	0.6036
	41–50	56.07 (10.79)	35.71–67.86	
	51–60	52.98 (11.83)	35.71–71.43	
	61–70	62.86 (6.96)	53.57–71.43	
	>70	57.86 (4.66)	53.57–64.29	
Psychological	<40	74.48 (11.66)	54.17–83.33	0.5889
	41–50	70.42 (8.88)	58.33–83.33	
	51–60	63.89 (12.55)	50–83.33	
	61–70	70.83 (8.33)	62.5–83.33	
	>70	69.17 (8.12)	58.33–79.17	
Social	<40	84.38 (22.47)	41.67–100	0.1907
	41–50	78.34 (15.81)	41.67–91.67	
	51–60	66.67 (21.73)	33.33–91.67	
	61–70	73.33 (20.75)	41.67–100	
	>70	88.33 (7.46)	83.33–100	
Environmental	<40	84.77 (13.09)	59.38–100	0.5217
	41–50	75.94 (12.76)	56.25–90.63	
	51–60	79.17 (11.64)	62.5–96.88	
	61–70	76.25 (13.9)	59.38–96.88	
	>70	81.88 (6.01)	75–90.63	

* *p*-value based on Kruskal–Wallis test, SD—standard deviation.

**Table 7:** Domain score for the acetabular and ring fracture group’s WHOQOL-BREF.

Domain or Item	Mean (SD) of Acetabular Group	Mean (SD) of the Pelvic Ring Group
Domains		
Physical health	13.38 (1.62)	13.06 (1.66)
Psychological	15.19 (1.79)	15.22 (1.62)
Social	16.33 (2.79)	16.55 (3.03)
Environmental	16.95 (1.85)	16.72 (1.91)

**Table 8:** The quality-of-life scales of the acetabular fracture group and comparisons across gender. *Physical functioning (PF); role limitations due to physical health (RP); pain (BP); general health (GH); energy/fatigue (VT); social functioning (SF); role limitations due to emotional problems (RE); emotional well-being (MH); physical health composite (PHC); mental health composite (MHC).*

	Age	PF	RP	BP	GH	VT	SF	RE	MH	PHC	MHC
Total		67.93 (25.05)	54.27 (46.06)	57.68 (26.31)	47.2 (4.62)	56.22 (20.12)	85.37 (27.51)	71.54 (41.87)	66.34 (17.98)	41.34 (9.49)	47.35 (10.91)
Range	29–86	0–100	0–100	0–100	40–60	10–100	0–100	0–100	16–92	20.46–56.25	14.35–60.66
Gender											
Women (*n* = 8)	51.25 (22.17)	62.5 (32.84)	37.5 (51.75)	48.44 (31.14)	47.5 (5.35)	45 (23.45)	64.06 (33.03)	54.17 (50.2)	58.5 (18.26)	38.02 (10.82)	39.98 (11.67)
Men (*n* = 33)	56.82 (14.7)	69.24 (23.22)	58.33 (44.49)	59.92 (25.04)	47.12 (4.51)	58.94 (18.61)	90.53 (23.81)	75.76 (39.33)	68.24 (17.66)	42.14 (9.14)	49.14 (10.1)
*p*-value ^a^	0.4012	0.7041	0.2277	0.2803	0.7203	0.0973	0.0166 *	0.2247	0.1218	0.3484	0.0352 *

^a^ Mann–Whitney U test. * indicates that there was statistical significance (*p* < 0.05).

**Table 9:** The statistical analysis of the acetabular group for the different age groups. *Physical functioning (PF); role limitations due to physical health (RP); pain (BP); general health (GH); energy/fatigue (VT); social functioning (SF); role limitations due to emotional problems (RE); emotional well-being (MH); physical health composite (PHC); mental health composite (MHC).*

	Age	PF	RP	BP	GH	VT	SF	RE	MH	(PHC)	(MHC)
<40	34.4 (4.58)	65 (26.03)	27.5 (32.17)	45.75 (26.95)	45.5 (4.38)	45 (22.24)	72.5 (37.17)	43.33 (41.72)	57.6 (24.53)	36.72 (8.78)	40.34 (14.35)
41–50	45.86 (3.3)	81.43 (15.7)	71.43 (41.9)	66.07 (28.5)	48.57 (6.9)	67.14 (18.22)	94.64 (14.17)	85.71 (37.8)	73.14 (8.23)	45.75 (8.37)	52.63 (5.8)
51–60	57.17 (1.8)	65.83 (37.34)	66.67 (51.64)	52.92 (34.84)	47.5 (4.18)	53.33 (22.51)	93.75 (15.31)	83.33 (40.82)	62 (21.28)	41.8 (11.88)	47.59 (8.94)
61–70	65 (2.96)	66.67 (18.54)	38.89 (48.59)	55.83 (17.18)	47.78 (3.63)	55.56 (16.48)	80.56 (30.05)	59.26 (49.38)	66.22 (14.16)	39.54 (8.29)	45.94 (9.63)
>70	76.89 (4.5)	63.33 (27.95)	77.78 (44.)	69.44 (23.78)	47.22 (4.41)	62.78 (17.7)	91.67 (25)	96.3 (11.11)	73.78 (13.87)	44.52 (9.74)	52.28 (8.93)
*p*-value ^a^		0.5314	0.1165	0.3441	0.7182	0.3343	0.1639	0.0517	0.3707	0.2134	0.0794
*p*-value ^b^		0.1506	0.0306 *	0.0519	0.3196	0.0863	0.0585	0.0069 *	0.1316	0.0279 *	0.0372 *

^a^ Kruskal–Wallis Test; ^b^ Mann–Whitney U test. * indicates that there was statistical significance (*p* < 0.05).

**Table 10:** Correlation between age and the scales of the SF-36 of the acetabular group. *Physical functioning (PF); role limitations due to physical health (RP); pain (BP); general health (GH); energy/fatigue (VT); social functioning (SF); role limitations due to emotional problems (RE); emotional well-being (MH); physical health composite (PHC); mental health composite (MHC).*

	PF	RP	BP	GH	VT	SF	RE	MH	PHC	MHC
R. Spearman Correlation	−0.085	0.22	0.249	0.072	0.207	0.215	0.294	0.231	0.193	0.271
*p*-value	0.5969	0.1673	0.1171	0.6563	0.1948	0.1761	0.0622	0.1468	0.2269	0.0867

**Table 11:** (**a**). Inter-scale correlations for acetabular group. (**b**). Spearman *p*-value for the inter-scale correlations of the acetabular group presented in (**a**). Physical functioning (PF); role limitations due to physical health (RP); pain (BP); general health (GH); energy/fatigue (VT); social functioning (SF); role limitations due to emotional problems (RE); emotional well-being (MH).

(**a**)								
**Scales**	**PF**	**RP**	**BP**	**GH**	**VT**	**SF**	**RE**	**MH**
PF	1	0.49	0.7	0.47	0.66	0.32	0.14	0.45
RP	0.49	1	0.65	0.43	0.42	0.64	0.67	0.38
BP	0.7	0.65	1	0.58	0.71	0.49	0.27	0.6
GH	0.47	0.43	0.58	1	0.61	0.41	0.12	0.37
VT	0.66	0.42	0.71	0.61	1	0.57	0.21	0.72
SF	0.32	0.64	0.49	0.41	0.57	1	0.52	0.48
RE	0.14	0.67	0.27	0.12	0.21	0.52	1	0.21
MH	0.45	0.38	0.6	0.37	0.72	0.48	0.21	1
(**b**)								
**Scales**	**PF**	**RP**	**BP**	**GH**	**VT**	**SF**	**RE**	**MH**
PF		0.0006	0.001	0.0001	0.001	0.0013	0.7394	0.001
RP	0.0006		0.001	0.0013	0.001	0.0002	0.001	0.0059
BP	0.001	0.001		0.001	0.001	0.0001	0.1552	0.001
GH	0.0001	0.0013	0.001		0.001	0.0137	0.2592	0.0028
VT	0.001	0.001	0.001	0.001		0.001	0.1867	0.001
SF	0.0013	0.0002	0.0001	0.0137	0.001		0.0119	0.001
RE	0.7394	0.001	0.1552	0.2592	0.1867	0.0119		0.1147
MH	0.001	0.0059	0.001	0.0028	0.001	0.001	0.1147	

**Table 12:** Mean (standard deviation) for age and scales of quality of life in the ring fracture group.

Characteristics of Patients (*n* = 34)	Mean (Standard Deviation)	Range
Age	51.09 (15.99)	22–79
Physical functioning (PF)	51.09 (15.99)	15–100
Role limitations due to physical health (RP)	72.79 (25.47)	0–100
Pain (BP)	49.26 (46.66)	10–100
General health (GH)	58.09 (23.23)	35–55
Energy/fatigue (VT)	45.44 (5.13)	25–100
Social functioning (SF)	57.79 (20.38)	0–100
Role limitations due to emotional problems (RE)	81.99 (26.86)	0–100
Emotional well-being (MH)	61.76 (45.05)	36–100
Physical health composite (PHC)	41.21 (9.19)	24.03–54.99
Mental health composite (MHC)	47.90 (10.76)	20.01–65.58

**Table 13:** Comparison between gender for scales and health composite of ring fracture group.

Gender	Female	Male	
count	10	24	
	Mean (standard deviation)		*p*-value ^a^
Age	56.3 (18.96)	48.92 (14.47)	
Physical functioning (PF)	58.5 (32.32)	78.75 (19.90)	0.1058
Role limitations due to physical health (RP)	40 (47.43)	53.13 (46.31)	0.3841
Pain (BP)	53.5 (26.36)	60 (22.12)	0.5543
General health (GH)	45 (4.08)	45.63 (5.58)	0.6935
Energy/fatigue (VT)	49 (18.83)	61.46 (20.24)	0.0950
Social functioning (SF)	73.75 (28.53)	85.42 (25.98)	0.1616
Role limitations due to emotional problems (RE)	46.67 (50.18)	68.06 (42.25)	0.2120
Emotional well-being (MH)	65.2911.32)	74.17 (15.06)	0.0588
Physical health composite (PHC)	38.06 (10.62)	42.52 (8.42)	0.3075
Mental health composite (MHC)	43.15 (9.94)	49.88 (10.65)	0.0587

^a^ Mann–Whitney U test.

**Table 14:** Statistical analysis of the different age groups of the ring fracture group. * Physical functioning (PF); role limitations due to physical health (RP); pain (BP); general health (GH); energy/fatigue (VT); social functioning (SF); role limitations due to emotional problems (RE); emotional well-being (MH).

	Age Group Division						
	<40	41–50	51–60	61–70	>70		
Scales and composites	Mean (standard Deviation)					*p*-value ^a^	*p*-value ^b^
PF	85 (17.53)	67.5 (26.9)	80.83 (22.23)	83 (19.87)	44 (23.29)	0.066	0.01501 *
RP	62.5 (46.29)	40 (51.64)	41.67 (46.55)	80 (32.6)	25 (43.3)	0.3344	0.0797
BP	54.06 (15.06)	55 (29.13)	56.25 (12.72)	83 (23.28)	48 (20.87)	0.1774	0.0333 *
GH	46.25 (4.43)	45.5 (4.97)	45 (7.07)	48 (4.47)	42 (4.47)	0.3978	0.0626
VT	58.75 (22.64)	56 (25.03)	51.67 (16.93)	67 (16.05)	58 (18.23)	0.6824	0.11815
SF	87.5 (23.15)	76.25 (29.73)	68.75 (40.12)	95 (6.85)	87.5 (17.68)	0.7318	0.42455
RE	83.33 (35.63)	46.67 (50.18)	61.11 (44.31)	66.67 (47.14)	53.33 (50.55)	0.5210	0.89342
MH	70 (11.9)	70.8 (17.79)	72 (18.76)	73.6 (10.04)	72.8 (14.81)	0.9502	0.65609
PHC	43.54 (8.02)	39.33 (9.91)	41.09 (8.4)	48.69 (7.7)	33.87 (7.66)	0.0815	0.0367 *
MHC	49.52 (9.03)	45.9 (13.32)	45.28 (13.79)	51.79 (6.74)	48.56 (9.2)	0.9375	0.53680

^a^ Kruskal–Wallis Test; ^b^ Mann–Whitney U test. * indicates that there was statistical significance (*p* < 0.05). and SF36 scale and composites labels as in Table 3.

**Table 15:** Correlation between age and the scales of the SF-36 of the ring fracture group. *Physical functioning (PF); role limitations due to physical health (RP); pain (BP); general health (GH); energy/fatigue (VT); social functioning (SF); role limitations due to emotional problems (RE); emotional well-being (MH).*

	PF	RP	BP	GH	VT	SF	RE	MH	PHC	MHC
R. Spearman Correlation	−0.242	−0.01	0.114	−0.07	0.068	0.039	−0.09	0.148	−0.001	0.061
*p*-value	0.1676	0.9542	0.5208	0.6924	0.7044	0.8277	0.6143	0.4035	0.9942	0.7328

**Table 16:** Inter-scale correlations for ring fracture group.

Scales	PF	RP	BP	GH	VT	SF	RE	MH
PF	1	0.6	0.61	0.51	0.37	0.36	0.57	0.44
RP	0.6	1	0.62	0.54	0.48	0.58	0.73	0.6
BP	0.61	0.62	1	0.49	0.52	0.43	0.5	0.66
GH	0.51	0.54	0.49	1	0.42	0.41	0.53	0.37
VT	0.37	0.48	0.52	0.42	1	0.64	0.55	0.78
SF	0.36	0.58	0.43	0.41	0.64	1	0.71	0.57
RE	0.57	0.73	0.5	0.53	0.55	0.71	1	0.62
MH	0.44	0.6	0.66	0.37	0.78	0.57	0.62	1

**Table 17:** Table of the Spearman *p*-value for the inter-scale correlations of the ring fracture group.

Scales	PF	RP	BP	GH	VT	SF	RE	MH
PF		0.0004	0.001	0.0038	0.0215	0.1313	0.0002	0.0107
RP	0.0004		0.0008	0.0015	0.0124	0.0012	0.001	0.0012
BP	0.001	0.0008		0.0064	0.0019	0.0108	0.0031	0.0001
GH	0.0038	0.0015	0.0064		0.0424	0.0286	0.0052	0.0644
VT	0.0215	0.0124	0.0019	0.0424		0.0001	0.0023	0.001
SF	0.1313	0.0012	0.0108	0.0286	0.0001		0.001	0.0001
RE	0.0002	0.001	0.0031	0.0052	0.0023	0.001		0.0003
MH	0.0107	0.0012	0.0001	0.0644	0.001	0.0001	0.0003	

## Data Availability

The data presented in this study are available on reasonable request from the corresponding author.

## Supplementary Materials

The following supporting information can be downloaded at: https://www.mdpi.com/article/10.3390/jcm14197036/s1, WHOQOL, SF36.

## References

[1] Van der Waal J.M., Terwee C.B., van der Windt D.A.W.M., Bouter L.M., Dekker J. (2005). The Impact of Non-Traumatic Hip and Knee Disorders on Health-Related Quality of Life as Measured with the SF-36 or SF-12. A Systematic Review. Qual. Life Res..

[2] White C.E., Hsu J.R., Holcomb J.B. (2009). Haemodynamically Unstable Pelvic Fractures. Injury.

[3] Ghosh S., Aggarwal S., Kumar V., Patel S., Kumar P. (2019). Epidemiology of Pelvic Fractures in Adults: Our Experience at a Tertiary Hospital. Chin. J. Traumatol..

[4] Ware J.E. (2000). SF-36 Health Survey Update. Spine.

[5] Skevington S.M., Lotfy M., O’Connell K.A., WHOQOL Group (2004). The World Health Organization’s WHOQOL-BREF Quality of Life Assessment: Psychometric Properties and Results of the International Field Trial. A Report from the WHOQOL Group. Qual. Life Res..

[6] Engelberg R., Martin D.P., Agel J., Obremsky W., Coronado G., Swiontkowski M.F. (1996). Musculoskeletal Function Assessment Instrument: Criterion and Construct Validity. J. Orthop. Res..

[7] Fugl-Meyer A.R., Bränholm I.-B., Fugl-Meyer K.S. (1991). Happiness and domain-specific life satisfaction in adult northern Swedes. Clin. Rehabil..

[8] Brazier J., Jones N., Kind P. (1993). Testing the Validity of the Euroqol and Comparing It with the SF-36 Health Survey Questionnaire. Qual. Life Res..

[9] Anglen J.O., Burd T.A., Hendricks K.J., Harrison P. (2003). The “Gull Sign”: A Harbinger of Failure for Internal Fixation of Geriatric Acetabular Fractures. J. Orthop. Trauma.

[10] Giannoudis P.V., Nikolaou V.S., Kheir E., Mehta S., Stengel D., Roberts C.S. (2009). Factors Determining Quality of Life and Level of Sporting Activity after Internal Fixation of an Isolated Acetabular Fracture. J. Bone Jt. Surg..

[11] Borg T., Berg P., Larsson S. (2012). Quality of Life after Operative Fixation of Displaced Acetabular Fractures. J. Orthop. Trauma.

[12] Suzuki T., Shindo M., Soma K., Minehara H., Nakamura K., Uchino M., Itoman M. (2007). Long-Term Functional Outcome after Unstable Pelvic Ring Fracture. J. Trauma.

[13] Banierink H., Reininga I.H.F., Heineman E., Wendt K.W., Ten Duis K., IJpma F.F.A. (2019). Long-Term Physical Functioning and Quality of Life after Pelvic Ring Injuries. Arch. Orthop. Trauma Surg..

[14] Banierink H., Ten Duis K., Wendt K., Heineman E., IJpma F., Reininga I. (2020). Patient-Reported Physical Functioning and Quality of Life after Pelvic Ring Injury: A Systematic Review of the Literature. PLoS ONE.

[15] Brouwers L., de Jongh M.A.C., de Munter L., Edwards M., Lansink K.W.W. (2020). Prognostic Factors and Quality of Life after Pelvic Fractures. The Brabant Injury Outcome Surveillance (BIOS) Study. PLoS ONE.

[16] Holstein J.H., Pizanis A., Köhler D., Pohlemann T., Working Group Quality of Life After Pelvic Fractures (2013). What Are Predictors for Patients’ Quality of Life after Pelvic Ring Fractures?. Clin. Orthop. Relat. Res..

[17] Verma V., Sen R.K., Tripathy S.K., Aggarwal S., Sharma S. (2020). Factors Affecting Quality of Life after Pelvic Fracture. J. Clin. Orthop. Trauma.

[18] Korovessis P., Baikousis A., Stamatakis M., Katonis P. (2000). Medium- and Long-Term Results of Open Reduction and Internal Fixation for Unstable Pelvic Ring Fractures. Orthopedics.

[19] Rommens P.M., Hessmann M.H. (2002). Staged Reconstruction of Pelvic Ring Disruption: Differences in Morbidity, Mortality, Radiologic Results, and Functional Outcomes between B1, B2/B3, and C-Type Lesions. J. Orthop. Trauma.

[20] Michaels A.J., Madey S.M., Krieg J.C., Long W.B. (2001). Traditional Injury Scoring Underestimates the Relative Consequences of Orthopedic Injury; discussion 396. J. Trauma.

[21] Miller A.N., Prasarn M.L., Lorich D.G., Helfet D.L. (2010). The Radiological Evaluation of Acetabular Fractures in the Elderly. J. Bone Jt. Surg..

[22] Ware J.E., Snow K.K., Kosinski M., Gandek B. (1993). SF-36 Health Survey: Manual and Interpretation Guide.

[23] Hays R.D., Shapiro M.F. (1992). An Overview of Generic Health-Related Quality of Life Measures for HIV Research. Qual. Life Res..

[24] Stewart A., Ware J.E., Stewart A., Sherbourne C.D., Ware J.E., Hays R.D., Wells K.B., Berry S.H., Kamberg C., Nelson E.C. (1992). Measuring Functioning and Well-Being: The Medical Outcomes Study Approach.

[25] Laucis N.C., Hays R.D., Bhattacharyya T. (2015). Scoring the SF-36 in Orthopaedics: A Brief Guide. J. Bone Jt. Surg..

[26] Oliver C.W., Twaddle B., Agel J., Routt M.L. (1996). Outcome after Pelvic Ring Fractures: Evaluation Using the Medical Outcomes Short Form SF-36. Injury.

[27] Lefaivre K.A., Slobogean G.P., Ngai J.T., Broekhuyse H.M., O’Brien P.J. (2014). What Outcomes Are Important for Patients after Pelvic Trauma? Subjective Responses and Psychometric Analysis of Three Published Pelvic-Specific Outcome Instruments. J. Orthop. Trauma.

[28] Borg T., Berg P., Fugl-Meyer K., Larsson S. (2010). Health-Related Quality of Life and Life Satisfaction in Patients Following Surgically Treated Pelvic Ring Fractures. A Prospective Observational Study with Two Years Follow-Up. Injury.

[29] Ponsford J., Hill B., Karamitsios M., Bahar-Fuchs A. (2008). Factors Influencing Outcome after Orthopedic Trauma. J. Trauma.

[30] Ayvaz M., Cağlar O., Yılmaz G., Güvendik G.I., Acaroğlu R.E. (2011). Long-Term Outcome and Quality of Life of Patients with Unstable Pelvic Fractures Treated by Closed Reduction and Percutaneous Fixation. Ulus. Travma Acil Cerrahi Derg..

[31] Höch A., Schneider I., Todd J., Josten C., Böhme J. (2018). Lateral Compression Type B 2-1 Pelvic Ring Fractures in Young Patients Do Not Require Surgery. Eur. J. Trauma Emerg. Surg..

[32] Hernefalk B., Eriksson N., Borg T., Larsson S. (2016). Estimating Pre-Traumatic Quality of Life in Patients with Surgically Treated Acetabular Fractures and Pelvic Ring Injuries: Does Timing Matter?. Injury.

[33] Patil A., Attarde D.S., Haphiz A., Sancheti P., Shyam A. (2021). A Single Approach for Management of Fractures Involving Both Columns of the Acetabulum: A Case Series of 23 Patients. Strateg. Trauma Limb Reconstr..

[34] Holbrook T.L., Hoyt D.B., Anderson J.P. (2001). The Importance of Gender on Outcome after Major Trauma: Functional and Psychologic Outcomes in Women versus Men. J. Trauma.

[35] Jaracz K., Kalfoss M., Górna K., Baczyk G. (2006). Quality of Life in Polish Respondents: Psychometric Properties of the Polish WHOQOL-Bref. Scand. J. Caring Sci..

[36] Hanestad B.R., Rustøen T., Knudsen O., Lerdal A., Wahl A.K. (2004). Psychometric Properties of the WHOQOL-BREF Questionnaire for the Norwegian General Population. J. Nurs. Meas..

[37] Australian WHOQoL Instruments: User’s Manual and Interpretation Guide.|Request PDF. https://www.researchgate.net/publication/289962910_Australian_WHOQoL_instruments_User's_manual_and_interpretation_guide.

[38] Nørholm V., Bech P. (2001). The WHO Quality of Life (WHOQOL) Questionnaire: Danish Validation Study. Nord. J. Psychiatry.

[39] Meena U.K., Sen R.K., Behera P., Tripathy S.K., Aggrawal S., Rajoli S.R. (2015). WHOQOL-BREF Hindi Questionnaire: Quality of Life Assessment in Acetabular Fracture Patients. IJOO.

[40] Bugajska J., JÍdryka-GÛral A. (2010). The Polish version of the SF-36v2 questionnaire for the quality of life assessment. Prz. Lek..

[41] Blanchard C.M., Côté I., Feeny D. (2004). Comparing Short Form and RAND Physical and Mental Health Summary Scores: Results from Total Hip Arthroplasty and High-Risk Primary-Care Patients. Int. J. Technol. Assess. Health Care.

[42] Rainer T.H., Yeung J.H.H., Cheung S.K.C., Yuen Y.K.Y., Poon W.S., Ho H.F., Kam C.W., Cattermole G.N., Chang A., So F.L. (2014). Assessment of Quality of Life and Functional Outcome in Patients Sustaining Moderate and Major Trauma: A Multicentre, Prospective Cohort Study. Injury.

[43] Failinger M.S., McGanity P.L. (1992). Unstable Fractures of the Pelvic Ring. J. Bone Jt. Surg..

[44] Kabak S., Halici M., Tuncel M., Avsarogullar L., Baktir A., Basturk M. (2003). Functional Outcome of Open Reduction and Internal Fixation for Completely Unstable Pelvic Ring Fractures (Type C): A Report of 40 Cases. J. Orthop. Trauma.

[45] Majeed S.A. (1989). Grading the Outcome of Pelvic Fractures. J. Bone Jt. Surg..

[46] Templeman D., Goulet J., Duwelius P.J., Olson S., Davidson M. (1996). Internal Fixation of Displaced Fractures of the Sacrum. Clin. Orthop. Relat. Res..

