# Supplementary material for: Evaluation of Patient-Assessed Quality of Life Questionnaires Following Operative Treatment of Pelvic Fractures
Source: J Clin Med. 2025 Oct 4;14(19):7036. doi: 10.3390/jcm14197036 (PMC12525433; doi:10.3390/jcm14197036)
Supplement: Supplementary file 1 [file jcm-14-07036-s001.zip › jcm-3870039-SF36.pdf]

Szanowni Państwo,

nadrzędnym celem wszelkich poczynań medycznych jest ratowanie życia i jego wydłużanie. Nie mniej ważnym jest czynienie tego życia lepszym, radośniejszym, dającym satysfakcję we wszelkich sferach dostępnych dla ludzkiej aktywności. W badaniach naukowych zwykło się to zawierać w pojęciu „jakość życia".

Myślę, że zainteresuje Państwa publikacja popularnego kwestionariusza oceny jakości życia SF-36 wraz z krótkim komentarzem prof. Jana Tylki, konsultanta krajowego w dziedzinie psychologii klinicznej.

Ponieważ, moim zdaniem, nie istnieje powszechnie akceptowany i dostępny sposób oceny jakości życia opracowany dla populacji polskiej, oczekuję na komentarze i propozycje dotyczące tego zagadnienia.

Ryszard Piotrowicz

Kwestionariusz oceny jakości życia SF-36 – wersja polska

Quality of life SF-36 questionnaire – the Polish version

Jan Tylka[1], Ryszard Piotrowicz[2]

[1] Instytut Psychologii, Uniwersytet Kardynała Stefana Wyszyńskiego, Warszawa

[2] Klinika i Zakład Rehabilitacji i Elektrokardiologi, Instytut Kardiologii, Warszawa

Kardiol Pol 2009; 67: 1166-1169

Metody oceny jakości życia można podzielić ze względu na:

1) kryterium administrowania (sposób stosowania):
   - pacjent sam dokonuje oceny (samoocena),
   - ankieter dokonuje oceny, prowadząc wywiad z badanym,

2) kryterium zawartości (zakres oceny):
   - ocena o charakterze ogólnym,
   - ocena szczegółowa (np. aktywność życia codziennego – szczegółowy indeks czynności).

W zależności od obszaru życia pacjenta, który jest przedmiotem zainteresowań badaczy, np. stan zdrowia fizycznego/psychicznego, aktywność ruchowa i wykonywanie czynności związanych z codziennym życiem, relacje z grupą społeczną, opracowywano stosowne metody badań.

Kwestionariusz SF-36 (*Short Form Health Survey*) ze względu na kryterium administrowania należy do metod samooceny. Pytania pozwalają na ocenę ośmiu wskaźników jakości życia tj.:

- funkcjonowanie fizyczne,
- ograniczenie w pełnieniu ról z powodu zdrowia fizycznego,
- dolegliwości bólowe,
- ogólne poczucie zdrowia,
- witalność,
- funkcjonowanie społeczne,
- ograniczenie w pełnieniu ról wynikające z problemów emocjonalnych,
- poczucie zdrowia psychicznego.

Wskaźniki można sumować w dwie skale – fizyczną i psychiczną, ale w naszej rzeczywistości jest to niemożliwe, gdyż brakuje norm dla populacji naszego kraju.

Różnice społeczne, kulturowe i pojęciowe pomiędzy populacjami różnych krajów są ogromne i dlatego należy zawsze dążyć do przystosowania narzędzia obcojęzycznego (w przypadku SF-36 – amerykańskiego) do specyfiki pojęć funkcjonujących w naszym języku i kulturze.

Należy pamiętać, że pytanie (jego treść) to rodzaj bodźca, który powoduje określoną reakcję osoby pytanej. Dlatego użyte w pytaniach pojęcia (nazwy lub opisy zjawisk) powinny odzwierciedlać specyfikę języka danej społeczności kulturowej.

W końcu lat 90. zeszłego wieku z inicjatywy firmy Servier w Instytucie Kardiologii przygotowano polską wersję językową kwestionariusza SF-36. Czterech psychologów dokonało niezależnego tłumaczenia, a następnie zweryfikowano jego trafność i sprawdzono funkcjonowanie

Adres do korespondencji:

prof. dr hab. n. med. Ryszard Piotrowicz, Instytut Kardiologii, ul. Alpejska 42, 04-628 Warszawa, tel.: +48 22 343 46 00,
e-mail: rpiotrowicz@ikard.pl

narzędzia w badaniu pilotażowym w grupach pacjentów kardiologicznych oraz chorych leczonych w Klinice Nerwic w Instytucie Psychiatrii i Neurologii w Warszawie.

Przyjęto, iż suma punktów w danym wskaźniku pokazuje jego pozytywną lub negatywną wartość, co oznacza, że można zastosować różnie skonfigurowaną punktację w zależności od tego, czy bardziej interesuje nas wysoki poziom pozytywnych ocen na temat zdrowia, czy poziom ocen negatywnych.

Zasada jest taka, że im wyższy wskaźnik, tym bardziej pozytywna jest samoocena badanego w zakresie tego, co zawierają przyjęte pojęcia jakości życia. Można też stosować zasadę, że im mniej negatywna jest samoocena badanego, a więc wskaźnik „negatywności” niższy, tym jakość życia jest lepsza.

Charakterystyka metody badawczej SF-36

Kwestionariusz jest przeznaczony do subiektywnej oceny stanu zdrowia. Składa się z 11 pytań zawierających 36 stwierdzeń, które pozwalają określić 8 elementów tj.: funkcjonowanie fizyczne, ograniczenia z powodu zdrowia fizycznego, odczuwanie bólu, ogólne poczucie zdrowia, witalność, funkcjonowanie socjalne, funkcjonowanie emocjonalne i zdrowie psychiczne.

Wskaźnik jakości życia jest sumą punktów oceny wszystkich 8 skal jakości życia i umożliwia ogólną ocenę stanu zdrowia. Według polskiej wersji kwestionariusza **najwyższa wartość punktowa** oznacza **najniższy stopień w ocenie jakości życia,** natomiast **najniższa wartość punktowa** oznacza **najwyższy poziom jakości życia.**

Wymiar fizyczny jakości życia – skale: I, II, IV, VIII; maksymalna liczba punktów: 103

Wymiar mentalny jakości życia – skale: III, V, VI, VII; maksymalna liczba punktów: 68

Indeks jakości życia – maksymalna liczba punktów: 171

Sposób liczenia polskiej wersji kwestionariusza SF-36 dostępny jest u autora.

Kwestionariusz oceny jakości życia SF-36

(wersja polska)

1. Generalnie możesz powiedzieć, że stan twojego zdrowia jest:

[ ] Doskonały
[ ] Bardzo dobry
[ ] Dobry
[ ] Zadowalający
[ ] Niezadowalający

2. Jak oceniasz stan swojego zdrowia w porównaniu z analogicznym okresem ubiegłego roku?

[ ] Dużo lepiej niż rok temu
[ ] Trochę lepiej teraz niż rok temu
[ ] Bardzo podobnie jak rok temu
[ ] Trochę gorzej niż rok temu
[ ] Dużo gorzej niż rok temu

3. Poniżej wymieniono w punktach czynności wykonywane zazwyczaj w ciągu dnia. Czy aktualnie Twoje zdrowie ogranicza Twoje możliwości ich wykonania? Jeżeli tak, to jak bardzo?

Czynności	Bardzo ogranicza	Trochę ogranicza	Nie ogranicza wcale
czynności wymagające energii, takie jak: bieganie, podnoszenie ciężarów, uczestniczenie w sportach wymagających dużego zaangażowania			
czynności o umiarkowanej trudności, takie jak: przesuwanie stołu, odkurzanie, gra w kręgle lub golfa			
podnoszenie lub dźwiganie zakupów			
pokonywanie kilku pięter schodów			
pokonywanie jednego piętra schodów			

schylanie się lub przyklękanie			
spacer dłuższy niż 1 km			
spacer ok. 500 m			
spacer ok. 100 m			
kąpiel lub ubieranie się			

4. Czy w ostatnim miesiącu miałeś(-aś) problemy z pracą lub codzienną aktywnością, które wynikały ze stanu zdrowia i powodowały:

	Tak	Nie
konieczność skrócenia czasu pracy lub innych czynności		
gorsze samopoczucie niż oczekiwałeś(-aś)		
ograniczenie w rodzaju pracy lub innych czynności		
wystąpienie utrudnień w wykonywanej pracy lub innych czynności		

5. Czy w ciągu ostatniego miesiąca miałeś(-aś) problemy związane z wykonywaną pracą lub codziennymi czynnościami wynikające z problemów emocjonalnych (np. poczucie depresji, zdenerwowanie)?

	Tak	Nie
skrócenie czasu wykonywanej pracy lub innej aktywności		
osiągnięcia (rezultaty) mniejsze, niż oczekiwałbyś(-abyś)		
niemożność wykonywania pracy lub innej czynności tak staranie jak zwykle		

6. Czy w ciągu ostatniego miesiąca twoje problemy zdrowotne lub emocjonalne miały wpływ na zwyczajne czynności, kontakty z rodziną, przyjaciółmi, sąsiadami lub innymi grupami?

[ ] Nie, wcale
[ ] Rzadko
[ ] Czasami
[ ] Nawet bardzo
[ ] Bardzo duży

7. Ile razy odczuwałeś(-aś) ból w ciągu ostatniego miesiąca?

[ ] Nigdy
[ ] Wyjątkowo
[ ] Bardzo rzadko
[ ] Często
[ ] Rzadko
[ ] Bardzo często

8. Jak często w ciągu ostatniego miesiąca ból zakłócał Twoją normalną pracę (zawodową i domową)?

[ ] Wcale
[ ] Trochę
[ ] Średnio
[ ] Nawet bardzo
[ ] Bardzo

9. Poniższe pytania dotyczą Twojego samopoczucia w ciągu ostatniego miesiąca. Na każde pytanie proszę udzielić jednej odpowiedzi najbardziej zbliżonej do stanu faktycznego. Ile razy wystąpił dany objaw w ciągu ostatniego miesiąca?

	Cały czas	Dużo czasu	Mało czasu	Większość czasu	Jakiś czas	Wcale
a) byłeś(-aś) pełen(-na) animuszu						
b) byłeś(-aś) bardzo zdenerwowany						
c) czułeś(-aś) się nic nie wart(-a) i nic nie było w stanie cię pocieszyć						
d) byłeś(-aś) wyciszony(-a) i spokojny(-a)						
e) byłeś(-aś) pełen(-na) energii						
f) byłeś(-aś) załamany(-a) i smutny(-a)						
g) czułeś(-aś) się zmarnowany(-a)						
h) byłeś(-aś) szczęśliwy(-a)						
i) byłeś(-aś) zmęczony(-a)						

10. Jak często w ciągu ostatniego miesiąca Twoje zdrowie fizyczne lub stan emocjonalny wpływały na kontakty towarzyskie (spotkania z rodziną i przyjaciółmi)?

[ ] Cały czas
[ ] Większość czasu
[ ] Część czasu
[ ] Mało czasu
[ ] Wcale

11. Jak bardzo prawdziwe lub fałszywe są według Ciebie poniższe stwierdzenia?

	Szczególnie prawdziwe	Czasami prawdziwe	Nie wiem	Czasami fałszywe	Szczególnie fałszywe
a) uważam, że stan mojego zdrowia jest lepszy niż innych osób, które znam					
b) jestem zdrowszy od innych osób, które znam					
c) przypuszczam, że stan mojego zdrowia ulegnie pogorszeniu					
d) stan mojego zdrowia jest doskonały					
