# Supplementary material for: Evaluation of Patient-Assessed Quality of Life Questionnaires Following Operative Treatment of Pelvic Fractures
Source: J Clin Med. 2025 Oct 4;14(19):7036. doi: 10.3390/jcm14197036 (PMC12525433; doi:10.3390/jcm14197036)
Supplement: Supplementary file 1 [file jcm-14-07036-s001.zip › jcm-3870039-WHOQOL.pdf]

SKRÓCONA WERSJA ANKIETY OCENIAJĄCEJ JAKOŚĆ ŻYCIA

THE WORLD HEALTH ORGANIZATION

QUALITY OF LIFE (WHOQOL) -BREF

The World Health Organization Quality of Life (WHOQOL)-BREF

© World Health Organization 2004

All rights reserved. Publications of the World Health Organization can be obtained from Marketing and Dissemination, World Health Organization, 20 Avenue Appia, 1211 Geneva 27, Switzerland (tel: +41 22 791 2476; fax: +41 22 791 4857; email: bookorders@who.int). Requests for permission to reproduce or translate WHO publications—whether for sale or for noncommercial distribution—should be addressed to Publications, at the above address (fax: +41 22 791 4806; email: permissions@who.int).

The designations employed and the presentation of the material in this publication do not imply the expression of any opinion whatsoever on the part of the World Health Organization concerning the legal status of any country, territory, city or area or of its authorities, or concerning the delimitation of its frontiers or boundaries. Dotted lines on maps represent approximate border lines for which there may not yet be full agreement.

The mention of specific companies or of certain manufacturers' products does not imply that they are endorsed or recommended by the World Health Organization in preference to others of a similar nature that are not mentioned. Errors and omissions excepted, the names of proprietary products are distinguished by initial capital letters.

The World Health Organization does not warrant that the information contained in this publication is complete and correct and shall not be liable for any damages incurred as a result of its use.

Acknowledgements

Translation of this document was performed on behalf of the World Health Organization by Dr Helena Baran-Furga, Dr Bogusłąw Habrat and Dr Karina Steinbarth-Chmielewska of the Institute of Psychiatry and Neurology, Warsaw, Poland, and Mr Leszek Śliwa.

WHOQOL-BREF – (SKRÓCONA WERSJA ANKIETY OCENIAJĄCEJ JAKOŚĆ ŻYCIA)

Kolejne pytania dotycza jakości Pana życia, zdrowia i innych dziedzin.
Przeczytam pytania oraz możliwe odpowiedzi. Proszę wybrać najbardziej właściwą odpowiedź. Jeśli nie jest Pan pewien, która z odpowiedzi jest właściwa, to proszę podać pierwszą o której Pan pomyślał, z zasady jest ona najbliższa prawdy. Proszę myśleć o swoim poziomie życia, nadziejach, przyjemnościach i troskach.

Zapytam Pana o sprawy życia z ostatnich czterech tygodni.

		Bardzo zła	Zła	Ani dobra, ani zła	Dobra	Bardzo dobra
1.	Jaka jest Pana jakość życia?	1	2	3	4	5

		Bardzo nie-zadowolony	Nie-zadowolony	Ani zadowolony, ani nie-zadowolony	Zadowolony	Bardzo zadowolony
2.	Czy jest Pan zadowolony ze swojego zdrowia?	1	2	3	4	5

Następne pytanie dotyczą nasilenia stanów, których Pan doznawał w ciągu 4 tygodni.

		Wcale	Nieco	Średnio	W dużym stopniu	W bardzo dużym stopniu
3.	Jak bardzo ból fizyczny przeszkadzał Panu robić to, co Pan powienien?	5	4	3	2	1
4.	W jakim stopniu potrzebuje Pan leczenia medycznego do codziennego funkcjonowania?	5	4	3	2	1
5.	Ile ma Pan radości w życiu?	1	2	3	4	5
6.	W jakim stopniu ocenia Pan, że Pana życie ma sens?	1	2	3	4	5
		Wcale	Nieco	Średnio	Dość dobrze	Bardzo dobrze
7.	Czy dobrze koncentruje Pan uwagę?	1	2	3	4	5
8.	Jak bezpiecznie czuje się Pan w swoim codziennym życiu?	1	2	3	4	5
9.	W jakim stopniu Pańskie	1	2	3	4	5

	otoczenie sprzyja zdrowiu?					

Poniższe pytania dotyczą tego jak Pan czuje się i jak się Panu wiodło w ciągu ostatnich

4 tygodni.

		Wcale	Nieco	Umiarkowa-nie	Przeważnie	W pełni
10.	Czy ma Pan wystarczająco energii w codziennym życiu?	1	2	3	4	5
11.	Czy jest Pan w stanie zaakceptować swój wygląd (fizyczny)?	1	2	3	4	5
12.	Czy ma Pan wystarczająco dużo pieniędzy na swoje potrzeby?	1	2	3	4	5
13.	Na ile dostępne są informacje, których może Pan potrzebować w codziennym życiu?	1	2	3	4	5
14.	W jakim zakresie ma Pan sposobność realizowania swoich zainteresowań?	1	2	3	4	5

		Bardzo źle	Źle	Ani dobrze ani źle	Dobrze	Bardzo dobrze
15.	Jak odnajduje się Pan w tej sytuacji?	1	2	3	4	5

		Bardzo nie-zadowolony	Nie-zadowolony	Ani za-dowolony ani nie-zadowolony	Zadowolony	Bardzo zadowolony
16.	Czy zadowolony jest Pan ze swojego snu?	1	2	3	4	5
17.	W jakim stopniu jest Pan zadowolony ze swojej wydolności w życiu codziennym?	1	2	3	4	5
18.	W jakim stopniu jest Pan zadowolony ze swojej zdolności (gotowości) do pracy?	1	2	3	4	5
19.	Czy jest Pan zadowolony z siebie?	1	2	3	4	5

20.	Czy jest Pan zadowolony ze swoich osobistych relacji z ludźmi?	1	2	3	4	5

21.	Czy jest Pan zadowolony ze swojego życia intymnego?	1	2	3	4	5
22.	Czy jest Pan zadowolony z oparcia, wsparcia, jakie dostaje Pan od swoich przyjaciół?	1	2	3	4	5
23.	Jak bardzo jest Pan zadowolony ze swoich warunków mieszkaniowych?	1	2	3	4	5
24.	Jak bardzo jest Pan zadowolony z placówek służby zdrowia?	1	2	3	4	5
25.	Czy jest Pan zadowolony z komunikacji (transportu)?	1	2	3	4	5

Poniższe pytanie odnosi się do częstotliwości doznań, jakich Pan doświadczał w okresie ostatnich 4 tygodni .

		Nigdy	Rzadko	Często	Bardzo często	Zawsze
26.	Jak często doświadczał Pana negatywnych uczuć, takich jak przygnębienie, rozpacz, lęk, depresja?	5	4	3	2	1

Czy ma Pan jakiś komentarz do tych pytań?

____

____

[Tę tabelę należy wypełnić po ukończeniu badania]

		Equations for computing domain scores	Raw score	Transformed scores*	
				4-20	0-100
27.	**Domain 1 Soma-Tyczna**	(6-Q3) + (6-Q4) + Q10 + Q15 + Q16 + Q17 + Q18<br>☐ + ☐ + ☐ + ☐ + ☐ + ☐ + ☐	a. =	b:	c:
28.	**Domain 2 Psycholo-Giczna**	Q5 + Q6 + Q7 + Q11 + Q19 + (6-Q26)<br>☐ + ☐ + ☐ + ☐ + ☐ + ☐	a. =	b:	c:
29.	**Domain 3 Socjalna**	Q20 + Q21 + Q22<br>☐ + ☐ + ☐	a. =	b:	c:

30.	**Domain 4**<br>**Środowis-ko**	Q8 + Q9 + Q12 + Q13 + Q14 + Q23 + Q24 + Q25<br>☐ + ☐ + ☐ + ☐ + ☐ + ☐ + ☐ + ☐	a. =	b:	c:

* Patrz: Procedures Manual, strony 17-19
